# The correlation between prenatal maternal active smoking and neurodevelopmental disorders in children: a systematic review and meta-analysis

**DOI:** 10.1186/s12889-023-15496-z

**Published:** 2023-03-30

**Authors:** Danrong Chen, Qing Niu, Shiping Liu, Wenchuan Shao, Yi Huang, Yifan Xu, Yihan Li, Jiani Liu, Xu Wang, Haibo Yang

**Affiliations:** 1 School of Public Health, Nanjing Medical University, Nanjing, 211166 China; 2 Department of Endocrinology, Children’s Hospital of Nanjing Medical University, Nanjing, 210008 China; 3 Department of Orthopedics, Children’s Hospital of Nanjing Medical University, Nanjing, 210008 China; 4 The First Clinical Medical College, Nanjing Medical University, Nanjing, 210029 China; 5 Department of Orthopedics, the First Affiliated Hospital of Nanjing Medical University, Nanjing, 210029 China; 6 Jiangsu Breast Disease Center, the First Affiliated Hospital of Nanjing Medical University, Nanjing, 210029 China; 7 Department of Emergency, Pediatric Intensive Care Unit, Children’s Hospital of Nanjing Medical University, Nanjing, 210008 China

**Keywords:** Maternal, Active smoking, Tourette syndrome, Chronic tic disorder, Developmental coordination disorder, Children

## Abstract

**Objective:**

To systematically evaluate the association between maternal active smoking during pregnancy and Tourette syndrome (TS), chronic tic disorder (CTD), and developmental coordination disorder (DCD) in children, and to provide evidence-based medical references to reduce the incidence of neurodevelopmental disorders in children.

**Method:**

We searched PubMed, Web of Science, Embase, and Cochrane Library to obtain relevant articles published before 4 August 2021. Two reviewers independently assessed the articles for eligibility and extracted data.

**Results:**

We included eight studies involving a total of 50,317 participants (3 cohort, 3 case–control, and 2 cross-sectional studies). The pooled effect estimates suggested that prenatal maternal active smoking is related to an increased risk of neurodevelopmental disorders (OR = 1.91, 95% CI: 1.30–2.80), especially DCD (OR = 2.25, 95% CI: 1.35–3.75). Maternal active smoking during pregnancy is not associated with TS (OR = 1.07, 95% CI: 0.66–1.73) in children.

**Conclusion:**

In this meta-analysis, we found evidence for a correlation between active smoking exposure in pregnant women and neurodevelopmental disorders in children. Owing to the differences in sample size, smoking categories and diagnostic methods, further research is needed to validate our results.

## Introduction

The adverse effects of prenatal maternal active smoking on birth outcomes have been a public health problem and have attracted extensive attention. Although an increasing number of people are aware of the serious harm of it, in some areas, the smoking rate among pregnant women is still high. The prevalence of maternal active smoking during pregnancy in Finland is approximately 15% [1]. Similar prevalence of 16% and 10.5% has been reported in studies conducted in Denmark and Canada, respectively [2, 3]. Moreover, prenatal maternal active smoking might relate to adverse birth outcomes, such as placenta abruption, miscarriage, being overweight and obese in childhood, and neurodevelopmental disorders [4–7].

A few studies have discussed the relationship between active smoking exposure during pregnancy and neurodevelopmental disorders in offspring. Neurodevelopmental disorders affect healthy growth and development in childhood, and may even lead to lifelong impairment. Tourette syndrome (TS) is a common childhood-onset, neurodevelopmental disorder characterised by multiple motor and vocal tics persisting for at least one year [8]. The prevalence of TS in children aged 6 – 15 is 0.77% [9], and morbidity of boys is significantly higher than girls [9, 10]. A few studies have indicated that TS may be associated with complex genetic factors; however, the aetiology has not been fully clarified [11, 12]. There are relatively few studies on the association between environmental factors and the risk of TS [13]. Children with TS tend to have several comorbid disorders, in particular chronic tic disorders (CTDs). CTD is a neurodevelopmental disorder that mainly manifests as chronic, fluctuating, motor, and/or phonic tics for more than 1 year [14]. Tics are reduplicative, abrupt vocalisations or movements that could be either simple or complex [15, 16]. Knight et al. reported a 2–3% prevalence of CTD, which occurs more commonly in boys than girls [9, 17]. Factors that contribute to CTD have not been completely confirmed. Developmental coordination disorder (DCD) is one of the most highly prevalent and chronic neurodevelopmental disorders, leading to grave consequences in children’s daily lives; the incidence of this disease in school-aged children is as high as 5–6% [18–20]. The chief manifestations of DCD are motor coordination difficulties and children being unable to reach the same level as their peers in the performance of fine and/or gross motor skills [21]. Moreover, 50–70% of children with DCD have motor coordination difficulties that persist into adolescence and even adulthood [18, 22]. Several factors are relevant to DCD, such as being born premature and being male [23]. Other risk factors are still controversial, and more studies are needed for further clarification.

In animal studies, evidence suggested that prenatal nicotine exposure substantially decreased cell size and cell layer thickness. Meanwhile, the proportion of medium-sized pyramidal neurons was reduced [24]. Omotoso et al. found that gestational nicotine exposure significantly altered neuronal morphology and elevated astrocytic count, which adversely affected neurodevelopment in rat offspring [25]. Previous research in human have implied that prenatal exposure to nicotine had a negative effect on the function of neurotransmitters and the development of the synapses [26]. Several studies have been performed to uncover the relationship between prenatal maternal smoking and the development of TS, CTD, and DCD in children, but the results were inconsistent. A few studies mentioned that prenatal maternal smoking was related to an increased risk of TS comorbid with CTD in offspring [27, 28]. However, in regard to TS only, this connection was not obvious [29–31]. There also appeared to be a discrepancy regarding the association between exposure to maternal smoking and the increased risk of DCD. Four studies suggested that prenatal maternal smoking was a risk factor for the development of DCD in children [32–35]. Other two studies without detailed definition of smoking and odds ratios have failed to detect this association [36, 37].

Therefore, we systematically reviewed the existing literature to determine the potential association between prenatal maternal active smoking and TS, CTD and CDC in offspring to provide epidemiological evidence to reduce the incidence of neurodevelopmental disorders. In addition, the positive relationship may encourage pregnant women to give up smoking.

## Materials and methods

### Search strategy

We performed our meta-analysis according to the Preferred Reporting Items for Systematic Reviews and Meta-analyses (PRISMA) guidelines [38]. Participants, Intervention, Comparison, and Outcome (PICO) search strategy was also applied in this study to identify eligible randomized controlled trials (RCTs) for inclusion (Participants—Children; Intervention – Active maternal smoking exposure; Comparison – Without active maternal smoking exposure; Outcomes – Neurodevelopmental disorders including TS, CTD, and DCD). Two reviewers (DC and QN) independently retrieved relevant articles from the online databases of PubMed, Web of Science, Embase, and Cochrane Library on 4 August 2021. The search terms we used were combinations of *maternal, prenatal, pregnancy, smoking, tobacco, cigarette, TS, CTD, and DCD.* We used the following search strategies:

1. (pregnancy) or (maternal) or (prenatal) or (gestational).
2. (smoking) or (tobacco) or (cigarette).
3. (Tourette syndrome) or (Tourette disorder) or (TS) or (chronic tic disorders) or CTD or (developmental coordination disorder) or DCD.
4. #1 AND #2 AND #3

We conducted the searches without any language restrictions. Grey literature from pre-print database was also retrieved comprehensively. In addition, we performed a manual search of all references in relevant studies to identify more studies and to avoid oversights. Consequently, the information from pertinent studies is comprehensive.

### Inclusion and exclusion criteria

The inclusion criteria were the following:

1. Epidemiological studies, including population-based cohort, case–control, and cross-sectional studies;
2. Data were recorded on the number of total participants, the number of cases, the ORs and corresponding 95% confidence intervals (CIs);
3. Maternal active smoking during pregnancy;
4. The incidence of TS, CTD, or DCD in children was reported.

The exclusion criteria for the studies were the following:

1. Review articles, conference abstracts, and animal experiments;
2. Studies without interested data on exposure and outcomes;
3. Studies that only recorded data on comorbidity;
4. Studies without corresponding 95% CIs.

### Study selection and data extraction

In accordance with the above inclusion and exclusion criteria, two reviewers (DC and QN) retrieved eligible articles by screening titles and abstracts. Additionally, they assessed full-text articles for further confirmation. They independently extracted relevant characteristics from each eligible article: the first author’s name, year of publication, research design, outcome(s), study population, % of males among cases, maternal age (years), area, diagnostic criteria, data source, time of exposure, smoking category, and adjustment variables.

### Quality assessment

Two independent reviewers (DC and SL) assessed the quality of the included literature, referring to the Newcastle Ottawa Scale (NOS, a custom designed scale for cohort/case–control studies) and the Agency for Healthcare Research Quality (AHRQ, a custom designed scale for cross-sectional studies) [39]. In terms of cohort and case–control studies, each article was assessed based on three broad perspectives: the selection process of the study subjects, comparability, and the assessment of exposure and outcomes. Eight items identify high-quality choices by using stars. Each item can be awarded no more than one star except for the ‘comparability’ category, which can be given at most two stars. A maximum of nine stars can be given to each article, and more stars indicate better quality. A study with no less than 7 stars is considered high-quality. With respect to cross-sectional studies, article quality was evaluated according to an 11-item checklist. When one of the items was met, the article was given one score. The overall scores, reflecting quality, were divided into three grades: 8–11 (high), 4–7 (moderate), and less than 3 (low) [40].

### Statistical analysis

Statistical analysis was processed in Stata/SE 12.0. We combined the ORs and corresponding 95% CIs from different studies, and applied random effects model to evaluate the correlation between prenatal maternal smoking and the risk of TS, CTD, and DCD in children. To explore the source of heterogeneity, we conducted a subgroup analysis. Sensitivity analysis was also performed in our study to assess the reliability of the included articles. Moreover, we further used Begg’s test, Egger’s test and funnel plot to examine the potential publication bias.

## Results

### Literature retrieval and overview of characteristics

**Fig. 1:**
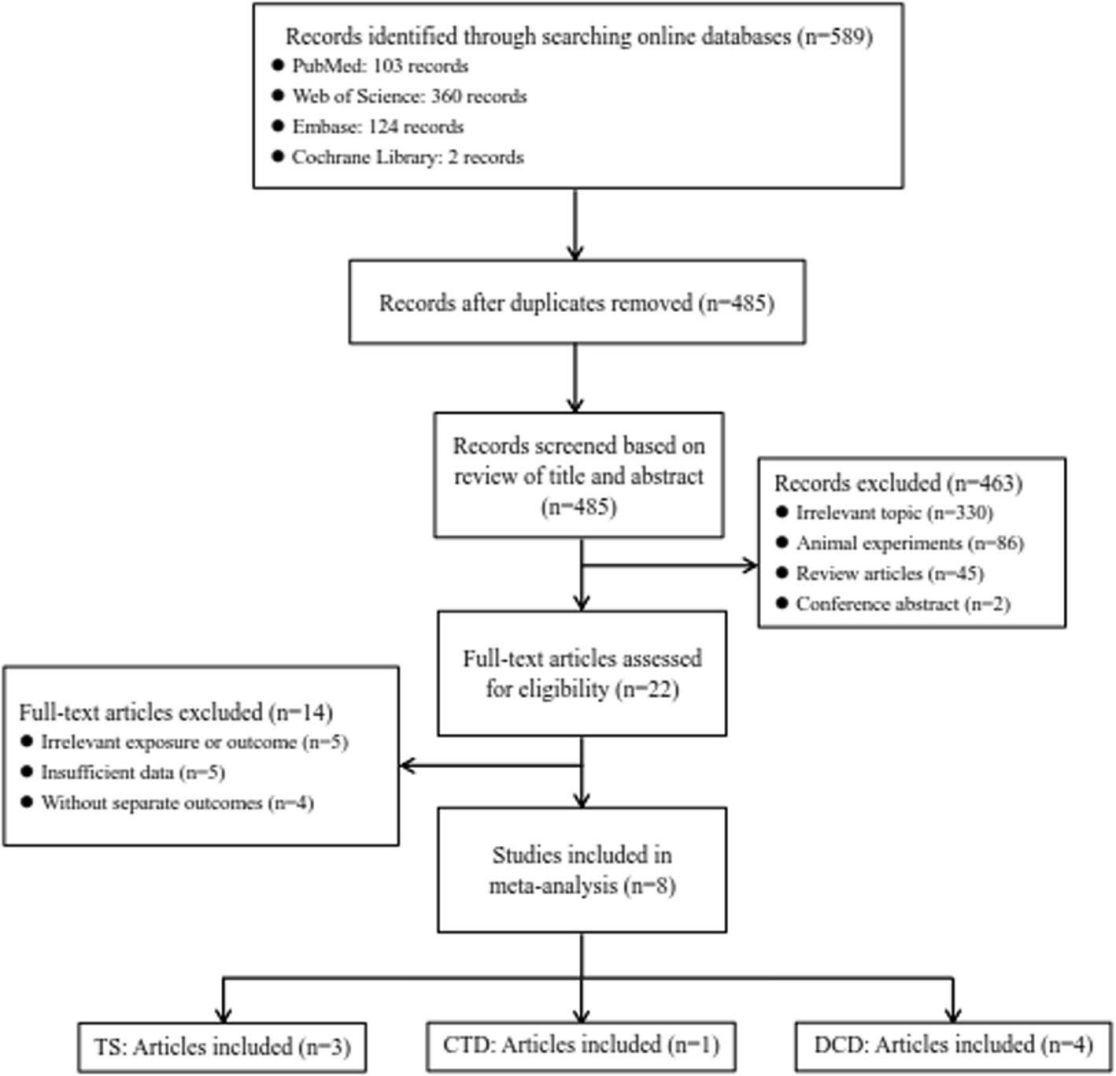
Flow diagram for the selection of eligible studies

We identified 589 articles in total by searching PubMed (103 records), Web of Science (360 records), Embase (124 records), and Cochrane Library (2 records). A total of 485 records were reserved after automatic duplicate removal. After reviewing the titles and abstracts, we further excluded 463 records, including 330 irrelevant research topics, 86 animal experiments, 45 review articles, and 2 conference abstracts. Moreover, 14 records on account of irrelevant exposure or outcomes, insufficient data, or no separate outcomes were excluded after the full-text review. Finally, we included 8 articles in our meta-analysis [29–35, 41] (Fig. 1).

Table 1 depicts the characteristics of these studies. Three were cohort studies, three were case–control studies (including two nested case–control studies), and the other two were cross-sectional. The sample sizes ranged from 122 to 33,354. Three of them explored the relationship between prenatal maternal smoking and TS; four investigated prenatal exposure to nicotine and DCD. Only one study discussed whether maternal smoking during pregnancy will increase the risk of CTD. All of the outcomes we concluded were separate diseases, and the comorbidity data were not recorded. Cohort and case–control studies were assessed referring to the NOS, which scored between 6 and 9. The quality of the two cross-sectional studies was evaluated using the AHRQ and scored 8 and 9.

**Table 1:** Characteristics of the studies included in this meta-analysis

The first author’s name	Year of publication	Research design	Outcome	Study population	% of males among cases	Maternal age (years)	Area	Diagnostic criteria	Data source	Time of exposure	Smoking category	Adjustment variables	Quality assessment score
Mathews, C. A	2014	Cohort	TS	No. of TS/total study population: 50/6,090	-	-	Avon,UK	DSM-IV-TR	Maternal questionnaires	In the last 2 months of pregnancy	Any smoking	Maternal age, parity, socioeconomic status level, pregnancy complications, alcohol and cannabis use in the last 2 months of pregnancy	9
Leivonen, S	2016	Nested case–control	TS	No. of maternal smoking/No. of controls: 356/2,698 No. of maternal smoking/No. of TS cases: 121/723	84.2	< 20y:72 20–29y:1,386 30–39y:1,169 ≥ 40y:71	Finland	ICD-10, ICD-9	A medical birth register	During pregnancy	Smoking during the first trimester only, and smoking throughout the pregnancy	Maternal and paternal psychiatric history, maternal and paternal age, birth weight, gestational age, and maternal socioeconomic status	8
Motlagh, M. G	2010	Case–control	TS	No. of maternal smoking/No. of controls: 1/62 No. of maternal smoking/No. of TS cases: 3/45	71.0	28.3 ± 4.1	Yale,USA	DSM-IV-TR	Maternal interview	During pregnancy	More than 10 cigarettes in 24 h at any point in the pregnancy	Gender, severe psychosocial stress, low birth weight, > 1 hypoxic event	6
Cubo, E	2014	Nested case–control	CTD	No. of maternal smoking/No. of controls: 21/89 No. of maternal smoking/No. of CTD cases: 25/64	70.0	30.41 ± 4.61	Spain	DSM-IV-TR	Birth certificates	For the entire pregnancy	Any smoking	Family history of tics, neonatal respiratory distress syndrome, body mass index, prenatal infection, and coexisting comorbid neuropsychiatric disturbances	7
Faebo Larsen, R	2013	Cohort	DCD	No. of DCD/total study population:1,001/33,354	-	< 25y:3,673 25–30y: 13,989 30–35y: 11,487 ≥ 35y: 4,205	Denmark	DCDQ’07	Maternal interview and the 7-year questionnaire	In the first trimester	Any smoking, 1 g– < 10 g/d, ≥ 10 g/d	Sex, gestational age, intrauterine growth restriction, maternal age, mother’s occupational status, alcohol consumption in the first trimester, and binge drinking in the first trimester	8
Christensen, L. H	2016	Cohort	DCD	1,023	-	25.18	Greenland and Ukraine	DCDQ’07	Structured interview questionnaire and telephone interviews	In the second or third trimester of pregnancy	Smokers were defined as women with serum cotinine concentrations > 10 ng/ml	Age of the mother and child, parity, sex, maternal educational level, maternal pre-pregnancy alcohol consumption and duration of breastfeeding	7
Yang, Q	2020	Cross-sectional	DCD	8586	61.5	19–25y: 237 26–30y: 1,870 31–35y: 3,858 36–48y: 1,621	Shanghai, China	MABC-2 screening combined with the paediatrician’s confirmation	A questionnaire interview	During pregnancy	Smoking at least one cigarette every day for at least six months	Prenatal exposure of secondhand smoke and breastfeeding time	9
Mahlberg, N	2019	Cross-sectional	DCD	122	59.3	-	Niagara, Canada	MABC-2	Parent questionnaires	During pregnancy	Either active or passive maternal smoke exposure	Age, sex, birth weight, premature birth, and household income	8

### Associations between prenatal maternal active smoking and neurodevelopmental disorders in children

**Fig. 2:**
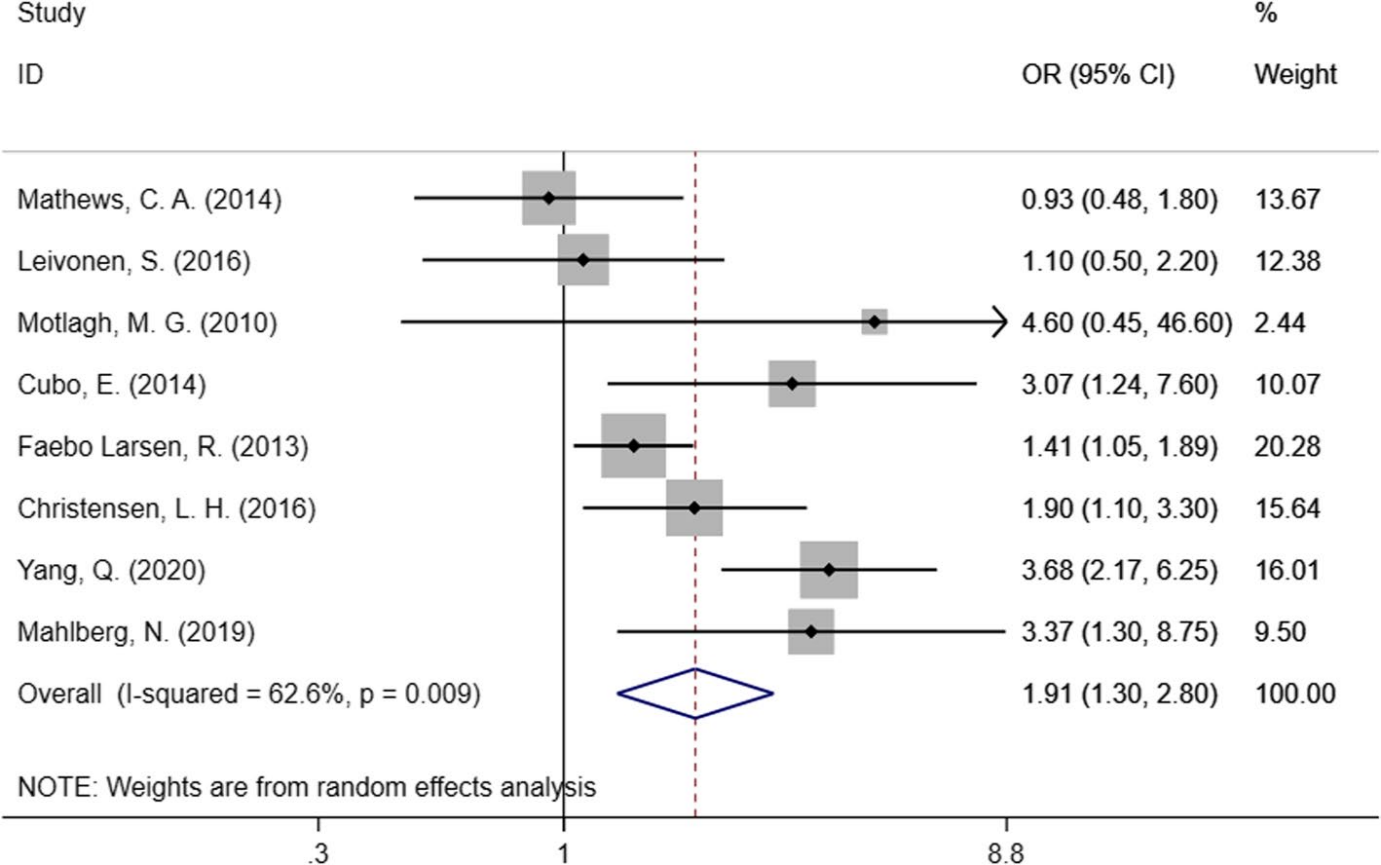
Forest plot of prenatal maternal smoking and neurodevelopmental disorders in children

We included eight studies in our meta-analysis to evaluate the relationship between prenatal maternal active smoking and the risk of neurodevelopmental disorders in offspring. As Fig. 2 shows, without considering the types of outcomes, prenatal maternal smoking accounted for the increased risk of three common neurodevelopmental disorders (TS, CTD, and DCD) in children, with a pooled effect estimate of 1.91 (95% CI: 1.30–2.80). Random-effects model was applied due to the remaining heterogeneity among the studies (I^2^ = 62.6%, *P* = 0.009).

### Associations between prenatal maternal active smoking and ts in children

**Fig. 3:**
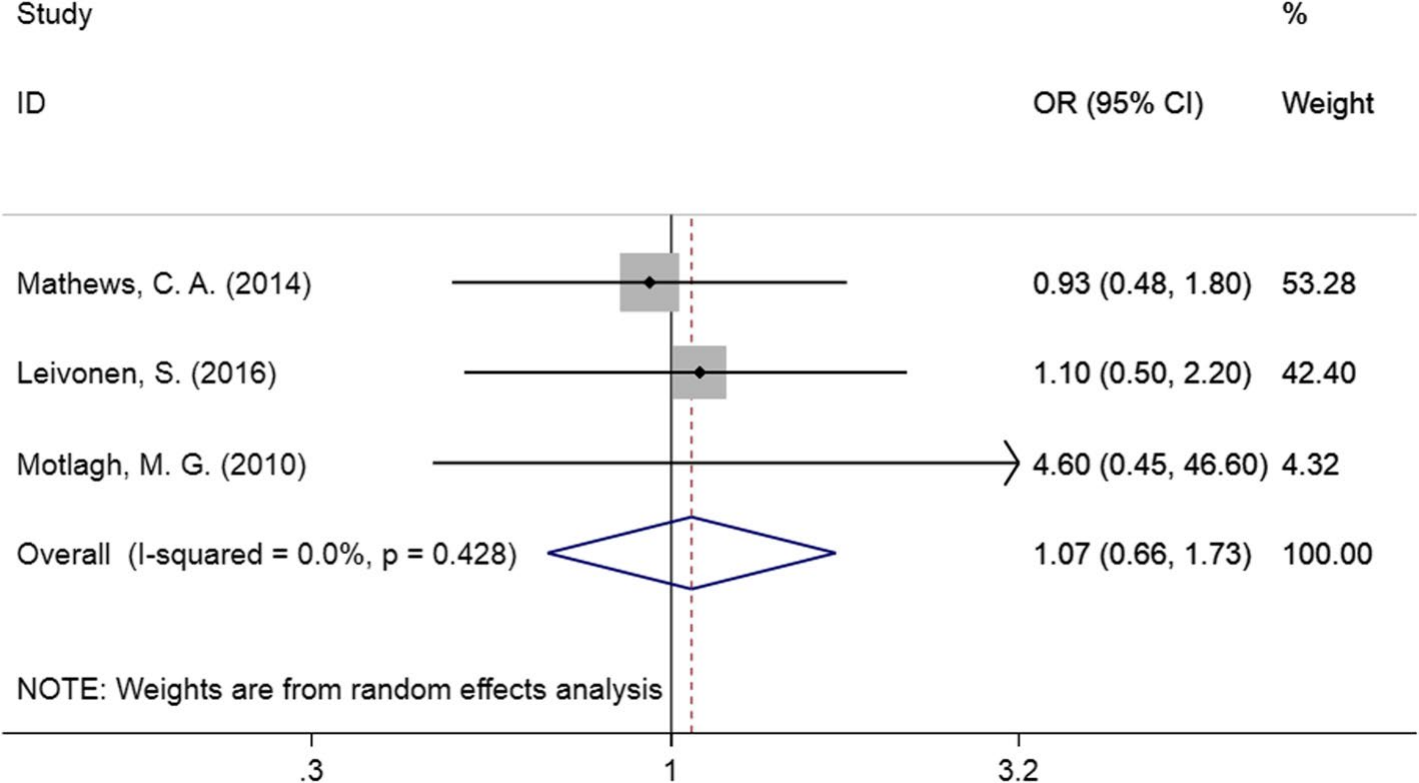
Forest plot of prenatal maternal smoking and TS in children

In our meta-analysis, we included three studies to assess the relationship between prenatal maternal active smoking and TS in children. As shown in Fig. 3, there was no statistically significant difference (OR = 1.07, 95% CI: 0.66–1.73, I^2^ = 0.0%, *P* = 0.428).

### Associations between prenatal maternal active smoking and ctd in children

To date, only one study has discussed the association between prenatal maternal active smoking and CTD in children (OR = 3.07, 95% CI: 1.24–7.60, *P* = 0.007) [41]. Therefore, forest plots cannot be drawn for analysis, and further research is needed.

### Associations between prenatal maternal active smoking and dcd in children

**Fig. 4:**
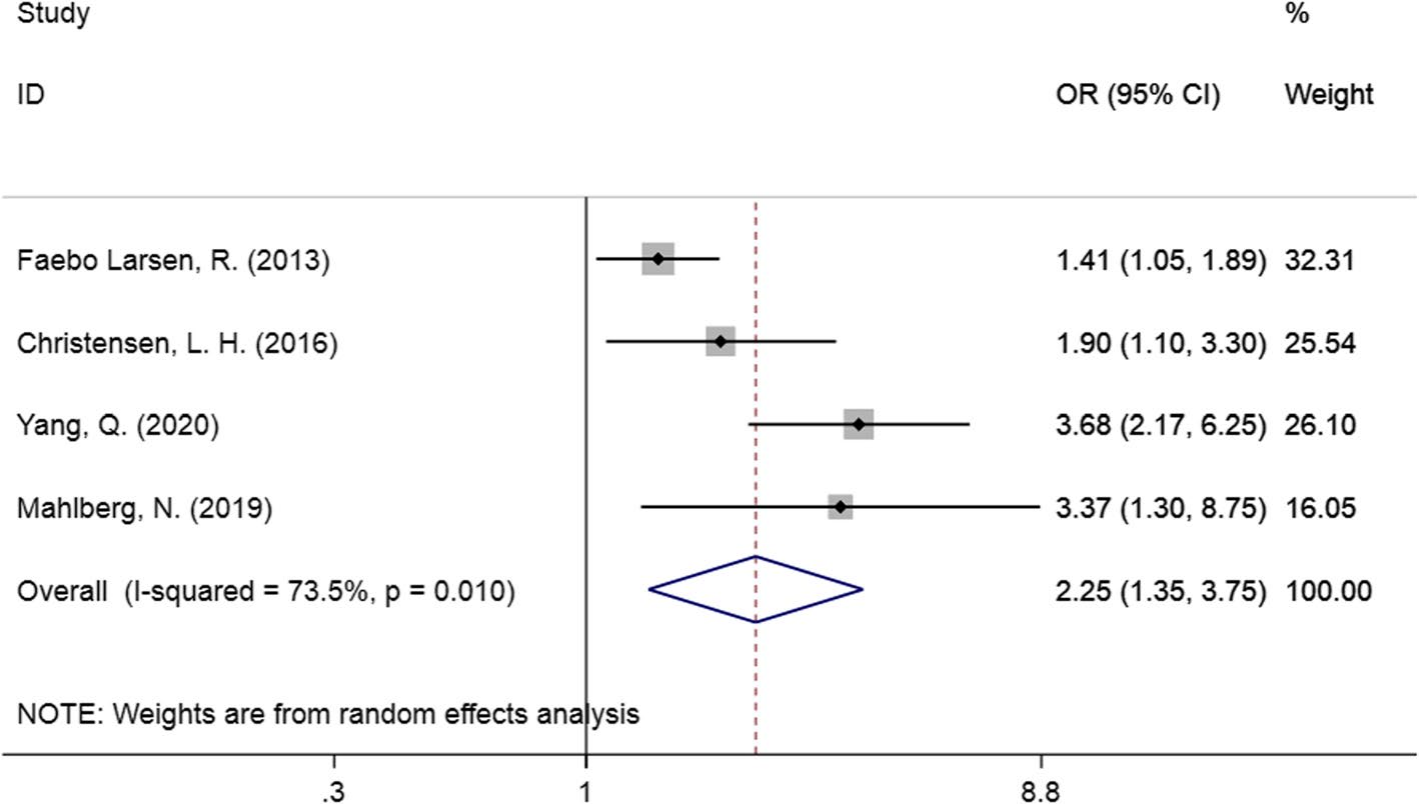
Forest plot of prenatal maternal smoking and DCD in children

Four studies examined the correlation between prenatal maternal active smoking and DCD in children. The forest plot is presented in Fig. 4 (OR = 2.25, 95% CI: 1.35–3.75, I^2^ = 73.5%, *P* = 0.010). As the findings indicated, prenatal maternal active smoking may increase the risk of DCD in offspring.

### Subgroup analysis

**Fig. 5:**
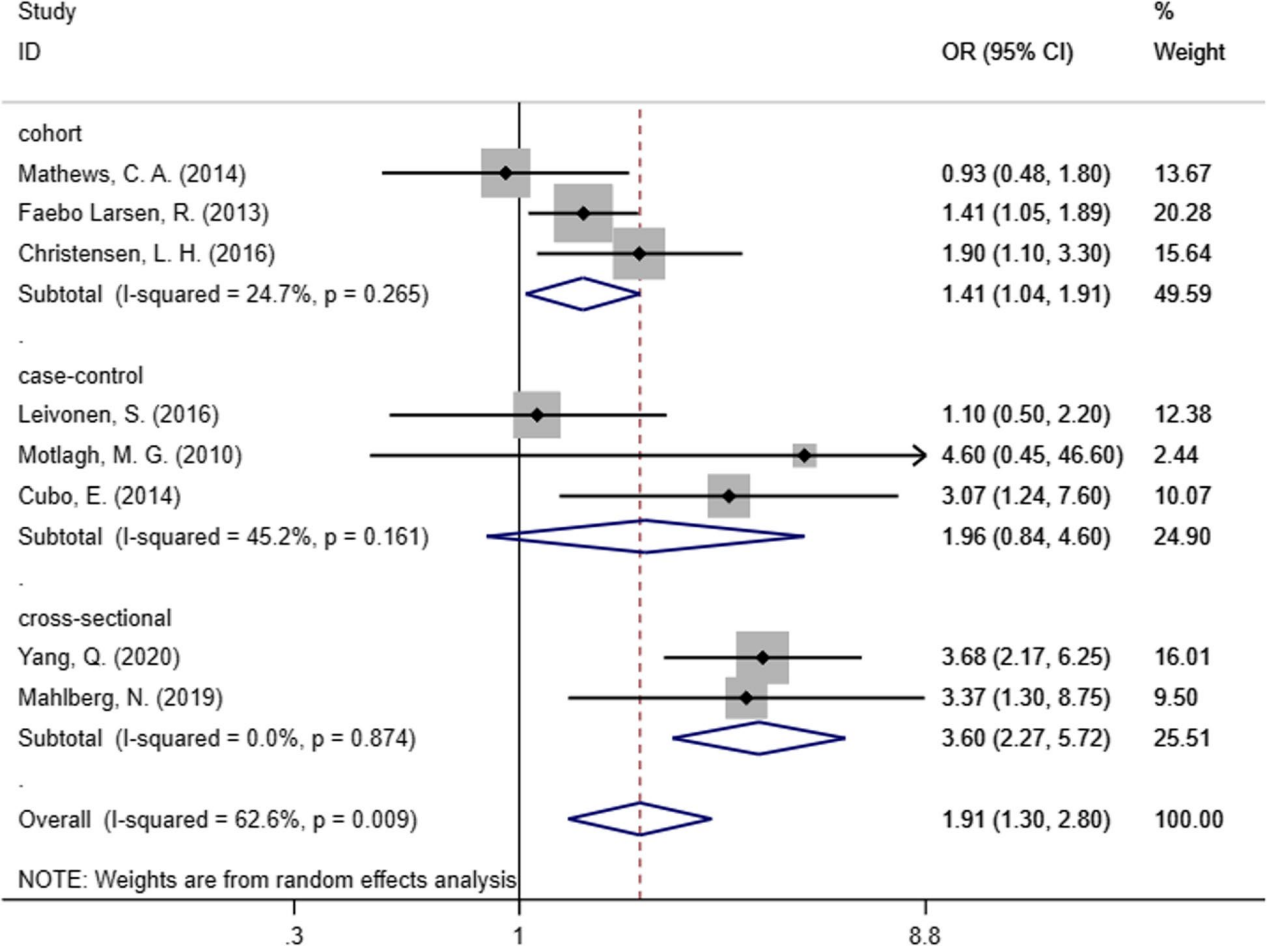
Subgroup analysis by research design

In our meta-analysis, we conducted a subgroup analysis based on the research design (cohort, case–control, and cross-sectional studies) in Fig. 5. The results suggested that the heterogeneity of cohort, case–control, and cross-sectional studies was reduced to 24.7% (*P* = 0.265), 45.2% (*P* = 0.161), and 0.0% (*P* = 0.874), respectively.

### Sensitivity analysis

**Fig. 6:**
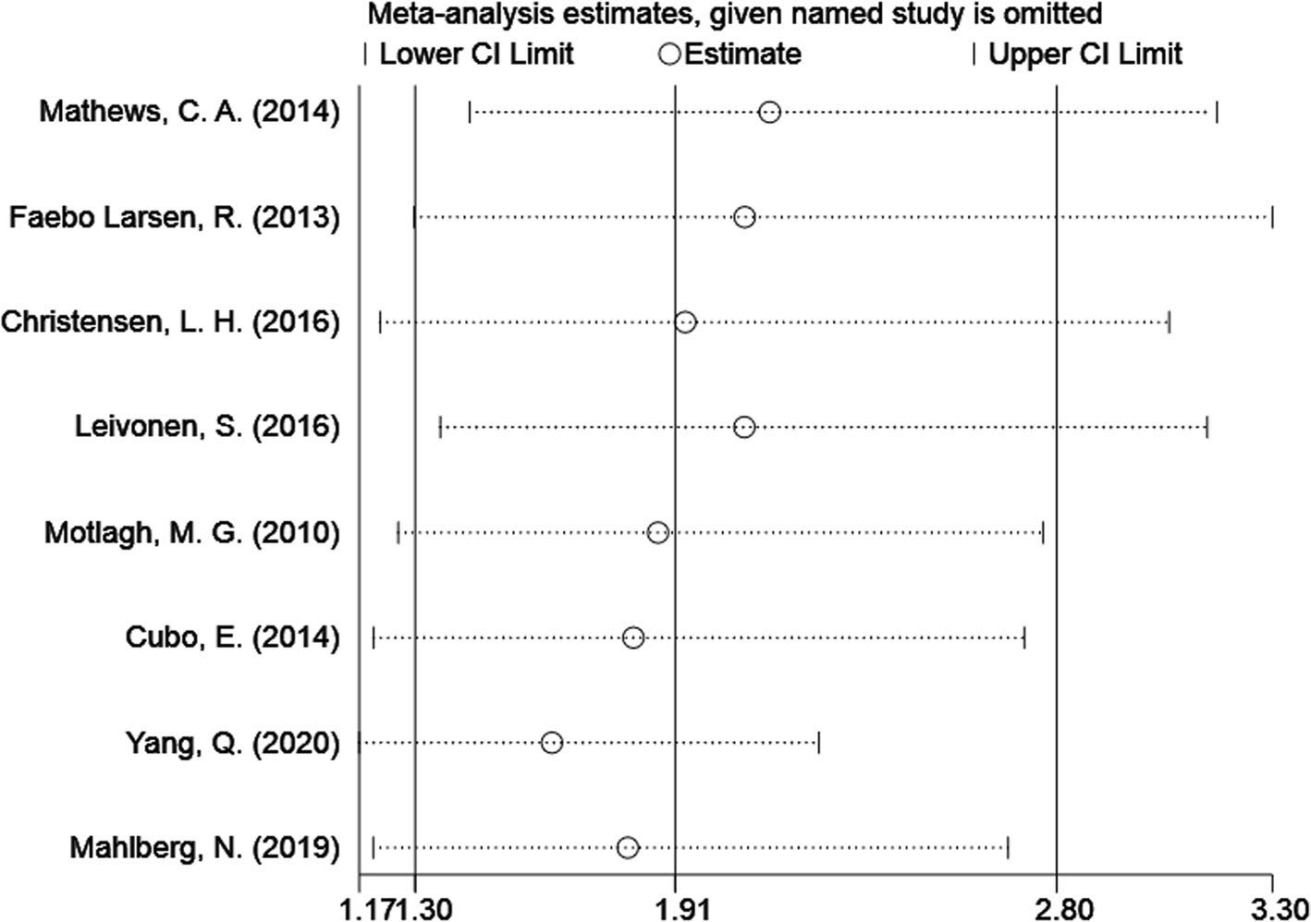
Sensitivity analysis of prenatal maternal smoking and neurodevelopmental disorders in children

Sensitivity analysis was further performed as part of our meta-analysis to assess the reliability of the included articles by excluding each article individually (Fig. 6). We did not find a significant difference when we excluded any study, which implies that the included studies are credible.

### Publication bias

**Fig. 7:**
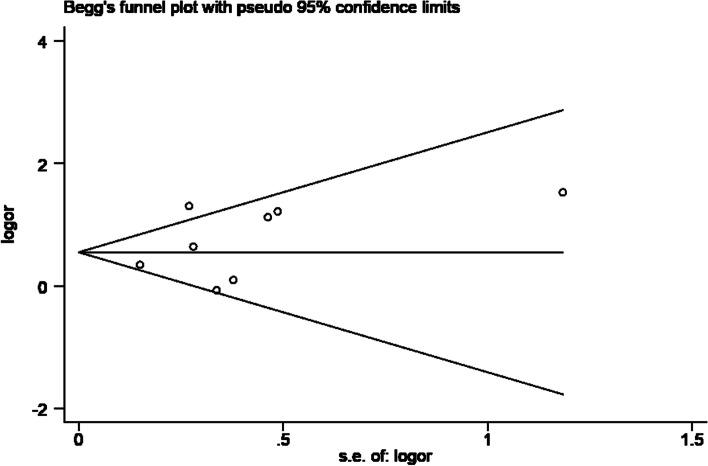
Funnel plot of prenatal maternal smoking and neurodevelopmental disorders in children

Ultimately, Begg’s test, Egger’s test and funnel plot were adopted to check whether publication bias existed in this study (Fig. 7). The funnel plot shows no obvious asymmetry. As Begg’s test (*P* = 0.536) and Egger’s test (*P* = 0.351) indicated, the results had no statistical significance, revealing that no visible publication bias exist among the included studies.

## Discussion

In this meta-analysis, we included eight studies to explore the relationship between prenatal maternal active smoking and the prevalence of TS, CTD, or DCD in children. According to the results, maternal active smoking during pregnancy contributes to a higher risk of neurodevelopmental disorders (OR = 1.91, 95% CI: 1.30–2.80). Three typical neurodevelopmental disorders were selected for research in this study. The effect estimate was 1.07 (95% CI: 0.66–1.73) in the TS group and 2.25 (95% CI: 1.35–3.75) in the DCD group. The results indicated that prenatal maternal active smoking is related to an increased risk of neurodevelopmental disorders, especially DCD. Maternal active smoking during pregnancy may also increase the risk of TS, but the relationship is not statistically significant. Further studies are needed to illustrate the relationship between maternal smoking during pregnancy and CTD due to the limited evidence from the existing research.

The relationship between maternal active smoking during pregnancy and neurodevelopmental disorders in children has been discussed for a long time, and the findings are controversial. Several studies have found prenatal maternal active smoking to be associated with the prevalence of TS and the increasing TS symptom severity [42, 43]. However, other studies discovered the opposite [29–31]. In regard to DCD, maternal smoking during pregnancy is often viewed as an associated factor. Two studies have reported this association, with precise indicators of maternal smoking [32, 33]. Nonetheless, articles without accurate measures of maternal smoking have not found this relevance [36, 37]. We combined the outcomes of TS, CTD and DCD in the forest plot to explore the relationship between maternal active smoking during pregnancy and these diseases. Based on the past findings, we classified all research results in different outcomes to obtain a better understanding of the relationship between maternal active smoking during pregnancy and TS, CTD, and DCD in children.

We performed subgroup analysis to reduce the significant heterogeneity in our meta-analysis. A few factors can explain the heterogeneity, including different diagnostic criteria for each disease, the time of exposure, maternal smoking category. First, different diagnostic criteria may cause the results to be slightly different. With regard to TS, two of the studies included used the DSM-IV-TR [29, 30], while the other one used the ICD-10 and the ICD-9 [31]. The DSM-IV-TR was also used by Cubo et al. to diagnose CTD [41]. In regard to DCD, DCDQ’07 was used by Larsen et al. and Christensen et al. [32, 33], while the other two studies diagnosed DCD primarily with MABC-2 [34, 35]. As the results indicated, children diagnosed using DCDQ’07 might have lower risk of DCD than children diagnosed according to MABC-2. Therefore, uniform diagnostic criteria for each neurodevelopmental disorders should be further adopted. All of the studies included the recorded time of exposure to maternal active smoking during the pregnancy; some of them had more detailed classifications such as the first, second, or third trimester. More research should be done to examine the differences in distinct exposure periods during pregnancy. Further, there is no unified standard in the classifications of maternal smoking. In some studies, maternal smoking was defined in detail, such as cigarette consumption per day and serum cotinine concentration. In other studies, any smoking was recorded as maternal smoking. It's worth noting that in Mahlberg et al.’s study, smoking was defined as either active or passive maternal smoke exposure, while in other studies only included maternal active smoking.

As the results indicated, active smoking exposure in pregnant women could increase the risk of DCD in children, but the association between maternal active smoking and TS in offspring is not significant. Nonetheless, the prevalence of TS in children may have a tendency to increase when women are actively exposed to smoking during pregnancy. Therefore, encouraging mothers to stop smoking, especially during pregnancy, should be necessary.

## Strengths and limitations

There are several strengths in this study; we review the current evidence to investigate whether maternal active smoking during pregnancy is related to neurodevelopmental disorders in children. In this systematic review and meta-analysis, we included eight studies that discuss the association between maternal active smoking and TS, CTD, and DCD in children, including cohort, case–control and cross-sectional studies, with a relatively large sample size. We conducted subgroup analysis to reduce significant heterogeneity.

We have to admit that this study has some limitations. First, different assessment tools of neurodevelopmental disorders was adopted, which may result in inaccurate diagnosis. Second, the objective biological measures for maternal smoking was lacked. More precise measurements of exposure should be used in further studies. Third, although we have adjusted the statistical data, information bias might still exist such as various areas and different maternal age in each study. Moreover, all of the studies we included are observational, which may cause innate bias.

## Conclusion

Active smoking exposure during pregnancy may increase the risk of several main kinds of neurodevelopmental disorders, especially DCD. Given the number of studies and sample size, further research is necessary to confirm these findings.

## Data Availability

All data related to the present study are available in the manuscript.
